# Supplementary material for: Collaborative Action of Microglia and Astrocytes Mediates Neutrophil Recruitment to the CNS to Defend against Escherichia coli K1 Infection
Source: Int J Mol Sci. 2022 Jun 11;23(12):6540. doi: 10.3390/ijms23126540 (PMC9223767; doi:10.3390/ijms23126540)
Supplement: Supplementary file 1 [file ijms-23-06540-s001.zip › ijms-1744600-supplementary.pdf]

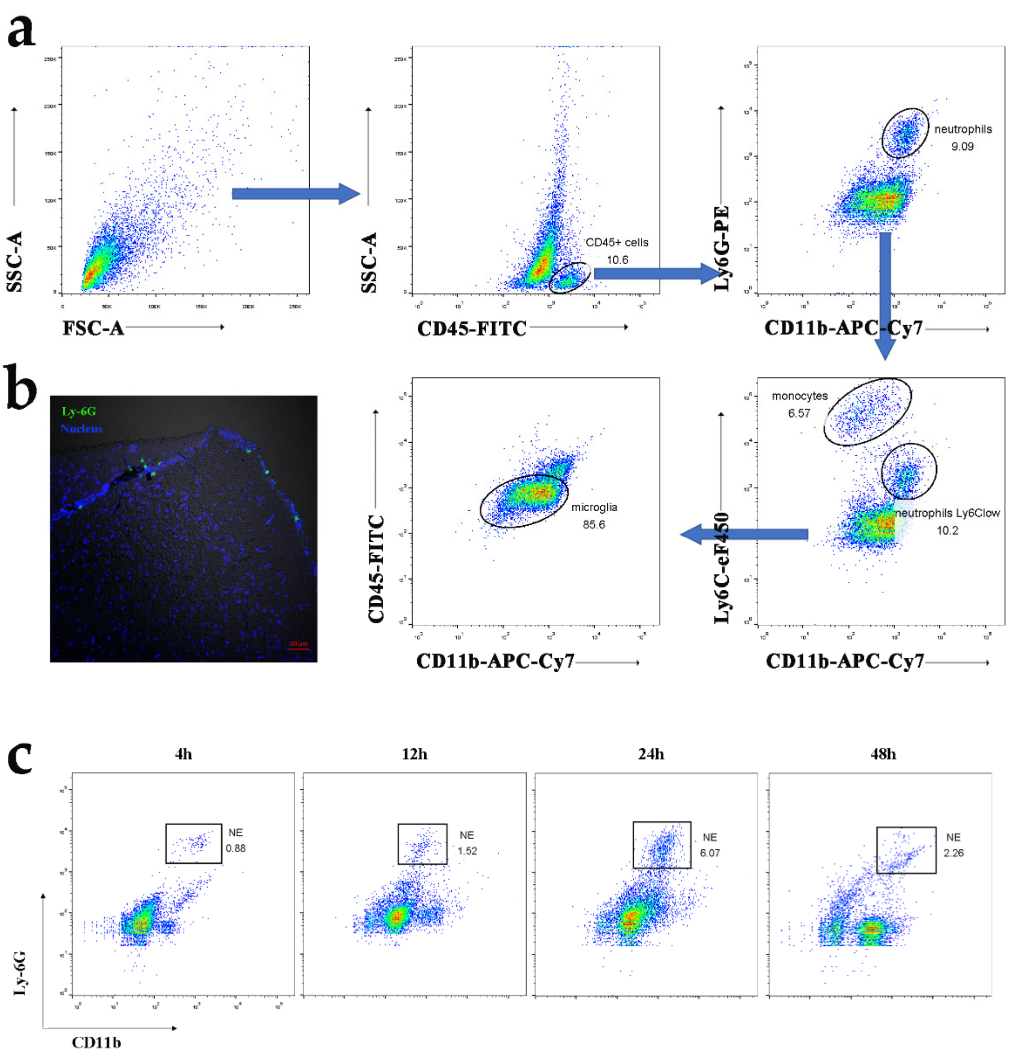

Figure S1. Neutrophils were recruited to CNS in response to *E. coli* K1 infection. (**a**) FACS (Fluorescence activated cell sorter) gating strategy used to identify neutrophils in the *E. coli* K1-infected mouse brain. (**b**) Confocal laser microscopy analysis of neutrophil migration in a neocortical region of *E. coli* K1-infected mouse brain. Scale bars, 50 μm. (**c**) Flow cytometric analysis of neutrophil counts in the mouse brain at 4, 12, 24 and 48 h after intravenous injection with *E. coli* K1.

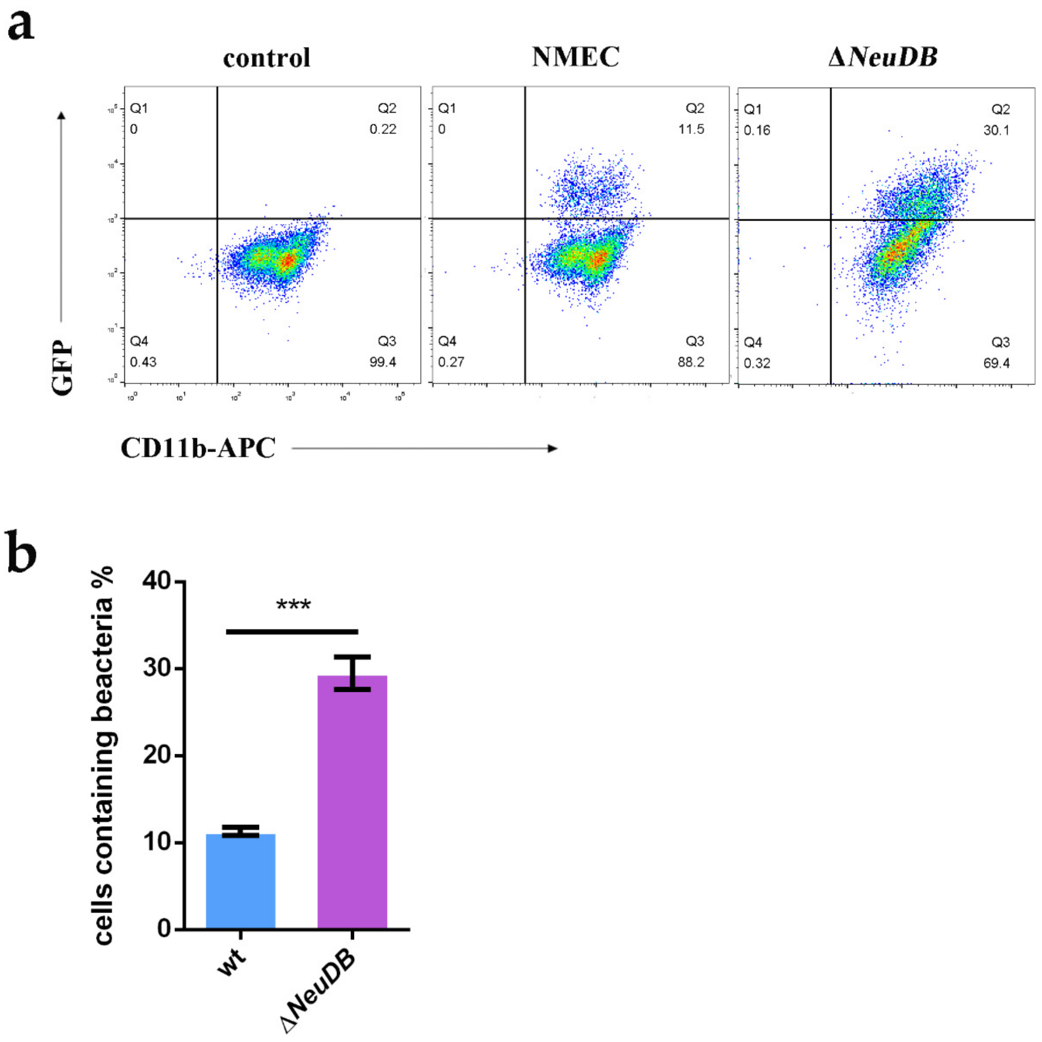

Figure S2. K1 capsule helps *E. coli* K1 to resist phagocytosis by microglia. (**a**,**b**) Flow cytometry analysis of microglia that contain bacteria when culturing microglia with *E. coli* K1 wild-type (wt) or K1 capsule-deficient strain (△*NeuDB*) *E. coli* K1. *** $p < 0.001$
